# Transcriptome Analyses Reveal Differential Transcriptional Profiles in Early- and Late-Dividing Porcine Somatic Cell Nuclear Transfer Embryos

**DOI:** 10.3390/genes11121499

**Published:** 2020-12-12

**Authors:** Zhiguo Liu, Guangming Xiang, Kui Xu, Jingjing Che, Changjiang Xu, Kui Li, Bingyuan Wang, Yulian Mu

**Affiliations:** Institute of Animal Sciences, Chinese Academy of Agricultural Sciences, Beijing 100193, China; zhiguoliu2010@126.com (Z.L.); m18813122758@163.com (G.X.); xukui2018xukui@163.com (K.X.); jingjing00xxx@163.com (J.C.); xcj950223@163.com (C.X.); likui@caas.cn (K.L.); mouyulian@caas.cn (Y.M.)

**Keywords:** pig, somatic cell nuclear transfer, RNA-seq, gene expression, embryo development

## Abstract

Somatic cell nuclear transfer (SCNT) is not only a valuable tool for understanding nuclear reprogramming, but it also facilitates the generation of genetically modified animals. However, the development of SCNT embryos has remained an uncontrollable process. It was reported that the SCNT embryos that complete the first cell division sooner are more likely to develop to the blastocyst stage, suggesting their better developmental competence. Therefore, to better understand the underlying molecular mechanisms, RNA-seq of pig SCNT embryos that were early-dividing (24 h postactivation) and late-dividing (36 h postactivation) was performed. Our analysis revealed that early- and late-dividing embryos have distinct RNA profiles, and, in all, 3077 genes were differentially expressed. Gene ontology (GO) and Kyoto Encyclopedia of Genes and Genomes (KEGG) analyses revealed that early-dividing embryos exhibited higher expression in genes that participated in the meiotic cell cycle, while enrichment of RNA processing- and translation-related genes was found in late-dividing embryos. There are also fewer somatic memory genes such as *FLRT2*, *ADAMTS1,* and *FOXR1*, which are abnormally activated or suppressed in early-dividing cloned embryos. These results show that early-dividing SCNT embryos have different transcriptional profiles than late-dividing embryos. Early division of SCNT embryos may be associated with their better reprogramming capacity, and somatic memory genes may act as a reprogramming barrier in pig SCNT reprogramming.

## 1. Introduction

Somatic cell nuclear transfer (SCNT) can be used to reprogram terminal differentiated somatic cells to a totipotency state and to generate a complete animal. Therefore, SCNT has been widely used in animal breeding, biological medicine, endangered species conservation, and germplasm protection [1]. However, reprogramming mediated by SCNT is a process that occurs in an uncontrolled manner. For large livestock, cloning efficiency remains low, and high rates of embryonic losses, still-births, and postnatal mortality have been typical outcomes [2]. Therefore, it is very important to elucidate the reprogramming barriers and explore effective methods to improve cloning efficiency in large livestock.

It has been reported that the timing of the first cleavage is an indicator of the subsequent developmental potential [3,4,5,6]. Embryos that complete the first cleavage sooner are more likely to form blastocysts and expanded blastocysts and to have a larger inner-cell mass [7]. It has also been demonstrated that embryo transfer of early-dividing embryos derived from in vitro fertilization (IVF) and intracytoplasmic sperm injection (ICSI) results in a higher pregnancy rate in humans [5,8]. In cattle [9], pigs [10,11], felines [3], and mice [12,13], it has been suggested that early-dividing embryos are more developmentally competent than those that cleave relatively later. In porcine SCNT, early-dividing embryos also have a higher blastocyst yield than late-dividing embryos [4,11,14]. However, the mechanisms underlying this phenomenon have not been fully elucidated.

To better understand the molecular mechanisms, the transcriptome of early-dividing (24 h postactivation) and late-dividing (36 h postactivation) pig SCNT embryos were analyzed by RNA-seq. Genes and pathways that may play key roles in SCNT embryo cleavage were analyzed.

## 2. Materials and Methods

All chemical reagents used in this study were purchased from Merck KGaA (Darmstadt, Germany) unless otherwise stated. The petri dishes used for the culture of oocytes and embryos were purchased from Thermo Fisher Scientific Co. (Waltham, MA, USA).

All experimental protocols related to animal work described in this study were reviewed and approved by the Institutional Animal Care and Use Committee (IACUC) of the Institute of Animal Sciences, Chinese Academy of Agricultural Sciences. All experiments were performed in accordance with the approved guidelines for animal care and management of research projects (IAS2020-25).

### 2.1. Somatic Cell Preparation

Pig embryonic fibroblast cells (PEFs) were established from a single fetus, following the protocol described by Lai et al. [15]. Isolated PEFs were frozen in liquid nitrogen. Before SCNT, PEFs were thawed and cultured for 2–3 days in Dulbecco’s modified Eagle’s medium (DMEM) (Thermo Fisher, Waltham, MA, USA) supplemented with 15% (*v*/*v*) fetal bovine serum (FBS) (Thermo Fisher, Waltham, MA, USA) at 38.5 °C in humidified air (100% humidity) containing 5% CO_2_. PEFs were then synchronized by the serum-starve method described by Lai et al. [15].

### 2.2. Oocyte Collection and In Vitro Maturation

Oocyte collection and in vitro maturation (IVM) were performed following the protocol described by us [16]. Briefly, porcine ovaries were obtained from a local slaughterhouse and transported to the laboratory within 3 h. Cumulus–oocyte complexes (COCs) were aspirated from the ovaries and matured in vitro for 42–44 h at 38.5 °C in humidified air (100% humidity) containing 5% CO_2_, as previously described [15]. Then, COCs were freed from cumulus cells by repeated pipetting in 0.1% hyaluronidase. Only matured oocytes with an extruded first polar body and uniform cytoplasm were used for somatic cell nuclear transfer.

### 2.3. Somatic Cell Nuclear Transfer (SCNT)

SCNT was performed as previously described [15]. Briefly, the first polar body and adjacent cytoplasm of matured oocytes were aspirated into an enucleation pipette, and a single PEF cell was injected into the perivitelline space and placed adjacent to the recipient cytoplasm. The resulting oocyte–donor cell complexes were placed in an electro-fusion medium for electrical activation by two successive DC pulses (1 s interval) at 1.2 kV/cm for 30 µs using a BTX ECM 2001 ElectroCell Manipulator. The complexes were then washed and incubated for 30 min in PZM-3 medium [17] before the fusion rate was evaluated using a stereomicroscope. With the abovementioned fusion medium (with 1.0 mM Ca^2+^), oocyte activation can be achieved during fusion. Fused oocyte–donor cell complexes were considered reconstructed embryos and were cultured in PZM-3 medium at 38.5 °C in humidified air (100% humidity) containing 5% CO_2_. The developmental status of the SCNT embryo was observed every 12 h, and cleaved 2-cell embryos were removed and cultured in a separate group for 6 days. Some of the 2-cell embryos collected at 24 h and 36 h postactivation were used for RNA sequencing.

### 2.4. SCNT 2-Cell Embryos Collection

SCNT 2-cell embryos cleaved at 24 h or 36 h postactivation were washed twice with PBS containing 0.1% BSA before being placed in 10× lysis buffer from a SMART-Seq™ v4 Ultra™ Low Input RNA Kit (Clontech, CA, USA) for RNA isolation.

### 2.5. RNA Isolation and Library Construction

RNA was isolated and amplified following the user manual of the SMART-Seq™ v4 Ultra™ Low Input RNA Kit for sequencing. Sequencing libraries were generated using the NEBNext^®^ Ultra™ DNA Library Prep Kit for Illumina^®^ (NEB, Ipswich, MA, USA), following the manufacturer’s recommendations. The RNA molecules that contained polyA were then sequenced on the Illumina HiSeq2500 platform.

### 2.6. Quality Control

Raw data (raw reads) of fastq format were first processed through in-house perl scripts. In this step, clean data (clean reads) were obtained by removing low-quality reads, reads containing adapters, and reads containing ploy-N from raw data. At the same time, Q20, Q30, and GC contents of the clean data were calculated. All downstream analyses were based on clean data. All clean RNA-seq data have been deposited in the SRA database under the BioProject accession number PRJNA683980.

### 2.7. Reads Mapping to Reference Genome

Pig reference genome (Sscrofa11.1) and gene model annotation files were downloaded from the Ensembl website (http://asia.ensembl.org/Sus_scrofa/Info/Index) directly. The index of the reference genome was built, and paired-end clean reads were aligned to the reference genome using STAR [18] (https://github.com/alexdobin/STAR/archive/2.5.2a.tar.gz).

### 2.8. Quantification of Gene Expression Level 

HTSeq (v0.6.1) was used to count the read numbers mapped to each gene. FPKM (fragments per kilobase of transcript sequence per million base pairs sequenced) of each gene was calculated based on the length of the gene and read counts mapped to this gene by using RSEM (v1.2.12). FPKM considers the effect of sequencing depth and gene length for the read count at the same time and is currently the most used method for estimating gene expression levels [19].

### 2.9. Analysis of Gene Differential Expression

Gene differential expression analysis of two groups (three biological replicates per condition) was performed using the DESeq2 R package. DESeq2 provides statistical routines for determining differential expression in digital gene expression data using a model based on negative binomial distribution. The resulting *p*-values were adjusted using Benjamini and Hochberg’s approach for controlling the false discovery rate (FDR). Genes with an FDR less than 0.05 and a twofold difference between the two groups found by DESeq2 were assigned as differentially expressed.

### 2.10. GO and KEGG Enrichment Analysis of Differentially Expressed Genes 

Gene Ontology (GO) and Kyoto Encyclopedia of Genes and Genomes (KEGG) enrichment analyses of differentially expressed genes were performed using the web-based gene set analysis toolkit WebGestalt [20] (http://www.webgestalt.org/). GO terms with a corrected *p*-value of less than 0.05 were considered significantly enriched by differential expressed genes. KEGG pathways with corrected *p*-values less than 0.05 were considered significantly enriched. GO and KEGG enrichment results were visualized using ggplot2 and ggpubr R packages.

## 3. Results

### 3.1. Dividing Time and Developmental Competence of SCNT Embryos

To compare the developmental competence of SCNT pig embryos that cleaved at 24 or 36 h postactivation, SCNT embryos were assessed visually every 12 h, and embryos that cleaved at 24 and 36 h were removed and placed in a separate group. Different groups were allowed to develop for 6 days in vitro. The data (Table 1) shows that the overall percentage of SCNT embryos that cleaved by 36 h postactivation was 68.1%. Of those embryos that cleaved within 36 h, 23.4% of them developed to blastocysts. Moreover, the proportion of SCNT embryos that cleaved by 24 h postactivation developed to blastocysts (33.6 ± 3.7%) was significantly higher than those that cleaved from 25 to 36 h postactivation (11.3 ± 3.5%). Similar results were observed by multiple research groups [4,11,14], indicating that early dividing SCNT embryos have better developmental competence.

### 3.2. Overview of the RNA-Seq Data in SCNT 2-Cell Embryos

**Figure 1:**
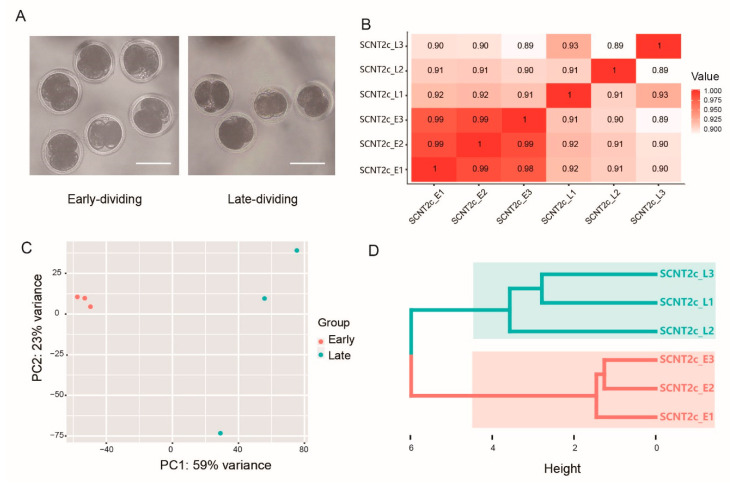
Morphology and global mRNA expression patterns of early-dividing and late-dividing SCNT 2-cell embryos. (**A**) The morphology of early-dividing (24 h postactivation) and late-dividing (36 h postactivation) SCNT embryos. The white bar represents 100 μm. (**B**) Heatmap of the Pearson correlation coefficient between each single embryo. (**C**) Principal component analysis of single embryos. (**D**) Unsupervised hierarchical clustering of single embryos.

To investigate the changes in gene expression between early- and late-dividing pig SCNT embryos, we collected six single porcine SCNT 2-cell embryos (Figure 1A): three of them cleaved at 24 h postactivation and the other three cleaved at 36 h postactivation, as described in Materials and Methods. Using the Illumina HiSeq 2500 sequencer, we generated 743 million raw reads from the six single embryos, with a read length of 125 bp. After strict quality control, more than 70 G clean bases were retained. The clean read data had an average Q30 of 90.33% and a GC content of 44.47% (Table S1). Between 85.20% and 95.97% of the clean reads could be mapped onto the Sscrofa11.1 genome (Table S2). FPKM was calculated as described in Materials and Methods. All genes expressed in at least one of the samples with FPKM > 0.1 were used in the downstream analysis (Table S3). Overall, 18,460 genes were identified in the six samples, which represented 71% of currently annotated 25,880 genes in Sscrofa11.1 pig reference genome.

The Human Genome Project recommends that under ideal sampling and test conditions, the Pearson correlation coefficient between samples in the same group should not be less than 0.95, which can be relaxed to 0.89 in specific project operations [21]. For all the genes we identified, the Pearson correlation coefficient between all samples was calculated using an R package. The Pearson correlation coefficient between embryos within the same group was equal to or higher than 0.89, indicating that the technical variation was reasonably low (Figure 1B). Unsupervised hierarchal clustering analysis and principal component analysis (PCA) revealed that early-dividing and late-dividing embryos formed distinct clusters (Figure 1C,D). Furthermore, it was also revealed that late-dividing SCNT 2-cell embryos were more different from each other than the early-dividing embryos (Figure 1C,D). By using the PACtools R package, we also identified genes most responsible for variation and retained genes that fell within the top and bottom 1% fractions of the PC1 and PC2 loadings’ range (Figure S1 and Table S4). Among these genes, the novel genes ENSSSCG00000037632 and ENSSSCG00000032623 were significantly and differentially expressed between the early-dividing group and the late-dividing group, indicating that they may play important roles in the process of somatic cell reprogramming.

### 3.3. Differentially Expressed Genes (DEGs) in Early-Dividing and Late-Dividing SCNT Embryos

**Figure 2:**
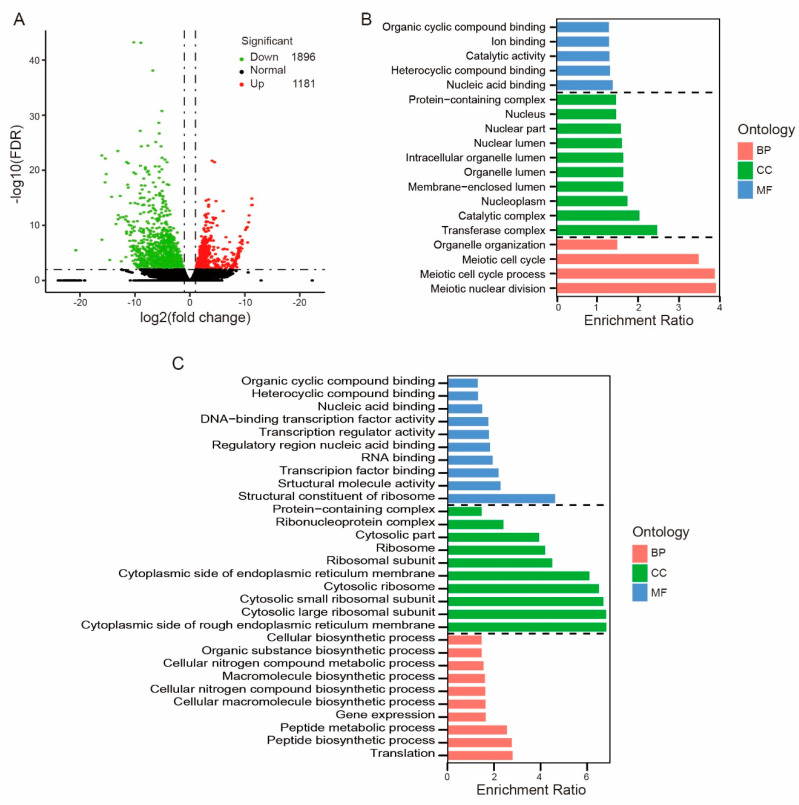
Differentially expressed genes (DEGs) and GO enrichment analysis. (**A**) Volcano plot for DEGs between early-dividing and late-dividing pig SCNT 2-cell embryos (FDR < 0.05 and |log2 (fold change)| >1); the upregulated genes (highly-expressed in early-dividing embryos) are represented by red dots and the downregulated genes (highly-expressed in late-dividing embryos) are represented by green dots. (**B**,**C**) GO functional enrichment analysis of the upregulated DEGs (**B**) and downregulated DEGs (**C**). The top 10 biological processes (BPs), cellular components (CCs) and molecular function (MF) terms were listed.

By using DESeq2, significant changes in gene expression between early-dividing and late-dividing groups (Figure 2A) were discovered. In total, 3077 genes were differentially expressed (FDR less than 0.05 and two-fold difference) between the early- and late-dividing groups, out of which 1896 genes were highly overexpressed in the late-dividing SCNT embryos, while 1181 were highly expressed in the early-dividing SCNT embryos (Figure 2A). This indicated that more genes were abnormally highly expressed in the late-dividing embryos, suggesting late-dividing embryos may have a higher total gene expression level compared to the early-dividing embryos.

### 3.4. GO and KEGG Analyses of DEGs in Early-Dividing and Late-Dividing SCNT 2-Cell Embryos

To explore the potential function of these DEGs, GO and KEGG databases were used. For GO classification, all DEGs of early-dividing vs. late-dividing embryos were divided into three ontologies: biological process (BP), cellular component (CC), and molecular function (MF). For upregulated genes, they were significantly enriched in terms of “meiotic cell cycle” and “organelle organization” of BP ontology (*p* < 0.05; Figure 2B); in terms “catalytic activity”, “heterocyclic compound binding”, “organic cyclic compound binding”, “ion binding”, and “nucleic acid binding” of MF ontology; of the 14 terms of CC ontology, the top 10 are listed in Figure 2B. Upregulated genes were mainly located in the nucleus, membrane-enclosed lumen, and protein-containing complex (Figure 2B).

Next, we performed GO enrichment analysis using the downregulated DEGs. We found that 87 terms were significantly enriched (Figure 2C), including 39 BP terms, 23 CC terms, and 25 MF terms. The top 10 terms of the three ontologies are shown in Figure 2. All these terms can be classified into transcription and translation. For example, in BP ontology, “peptide biosynthetic/metabolic process”, “cellular macromolecule biosynthetic process”, and “cellular nitrogen compound metabolic process” were enriched. In CC ontology, “ribosome”, “ribosomal subunit”, “rough endoplasmic reticulum membrane”, and “ribonucleoprotein complex” were enriched. These terms indicated that downregulated DEGs were involved in translation and amino acid biosynthesis or metabolisms. At the same time, transcription and RNA processing terms were highly enriched, especially “gene expression” in BP ontology and “transcription factor binding” and “regulatory region nucleic acid binding“ in MF ontology. These terms indicate that downregulated DEGs also participate in the regulation of transcription and gene expression. To summarize the GO analysis results, we found that early-dividing SCNT embryos have higher expression levels of genes that participate in the meiotic cell cycle, while genes that participate in transcription and translation, especially in the ribosome, were highly expressed in late-dividing SCNT embryos.

**Figure 3:**
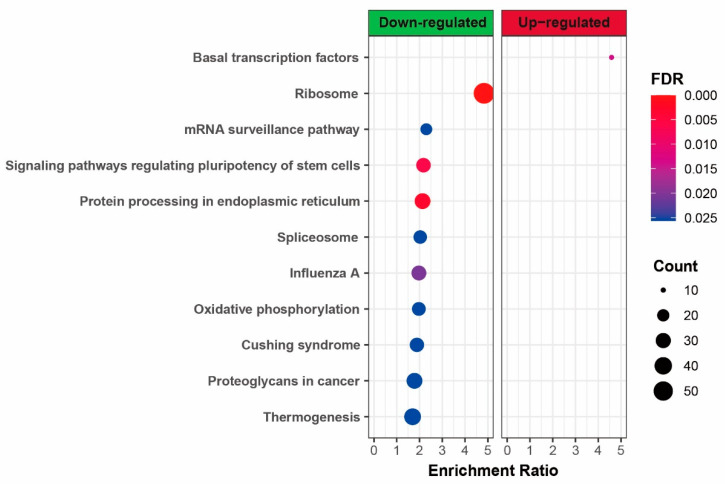
KEGG pathway enrichment analysis of upregulated and downregulated DEGs. The top 10 KEGG pathways that were significantly enriched for downregulated DEGs are listed. Only one pathway was enriched for upregulated DEGs.

**Figure 4:**
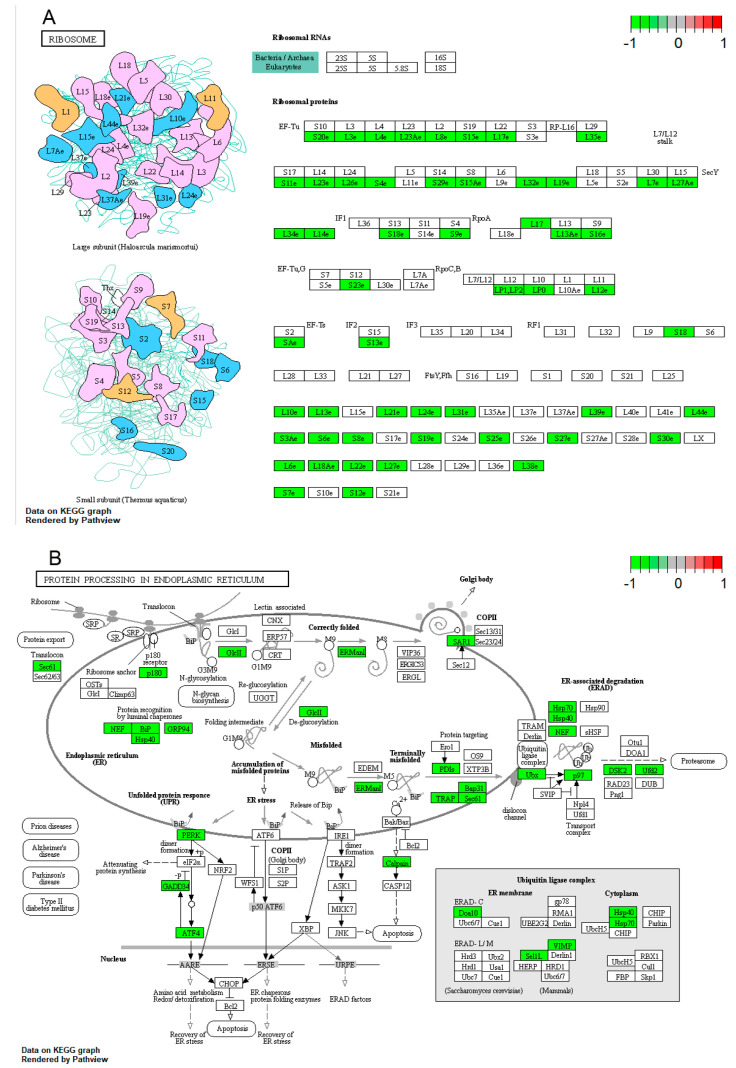
The KEGG pathways of ribosome (**A**) and protein processing in the endoplasmic reticulum (**B**). Genes highlighted in green are enriched and highly expressed in late-dividing SCNT embryos.

The upregulated and downregulated DEGs were separately mapped to KEGG pathways; the top 10 significantly (FDR < 0.05) enriched pathways are listed in Figure 3. The genetic information processing pathway is the largest classification in the result of KEGG analysis, including “ribosome”, “mRNA surveillance pathway”, “protein processing in endoplasmic reticulum”, “spliceosome”, and “basal transcription factors”. Among these pathways, only “basal transcription factors” were significantly enriched for the upregulated genes, while the other four pathways were significantly enriched for the downregulated genes. As illustrated in Figure 4, a lot of genes that participated in “ribosome” and “protein processing in endoplasmic reticulum” pathways were highly expressed in late-dividing SCNT embryos, especially the genes that participated in the large subunit of the ribosome.

Furthermore, “signaling pathways regulating pluripotency of stem cells” are involved in cellular community classification. For metabolism classification, only ‘oxidative phosphorylation’ was involved. For organismal system classification, the ‘thermogenesis’ pathway was identified. Importantly, three pathways of “influenza A”, “Cushing syndrome”, and “proteoglycans in cancer” were involved in human disease classification. All genes involved in these pathways were highly expressed in the late-dividing SCNT embryos, but not in early-dividing embryos.

GO and KEGG analyses provide an overview of the function of DEGs. In early-dividing embryos, the meiotic cell cycle and basal transcription factor genes were highly expressed. Conversely, not only were the genes related to mRNA surveillance and spliceosome, ribosome, and protein processing, the genes involved in human disease classification were also highly expressed in late-dividing embryos, implying that in late-dividing embryos, the regulation of gene expression is disordered and the genes that are not involved in embryonic development are abnormally activated.

### 3.5. Oocyte Quality-Related Genes in SCNT Embryo Dividing

Several studies have suggested that IVF [3,10,12], ICSI [22] embryos, as well as parthenogenetic embryos that cleave relatively earlier, are more likely to develop into the blastocyst stage in vitro [8]. It has been confirmed by multiple research groups [4,11,23] that early-dividing pig SCNT embryos are more successful at developing in culture than late-dividing embryos. As this phenomenon is true in IVF, ICSI, SCNT, and parthenogenetic embryos, it has been suggested that the major mechanistic components responsible for developmental potential are the intrinsic quality of oocytes. 

**Figure 5:**
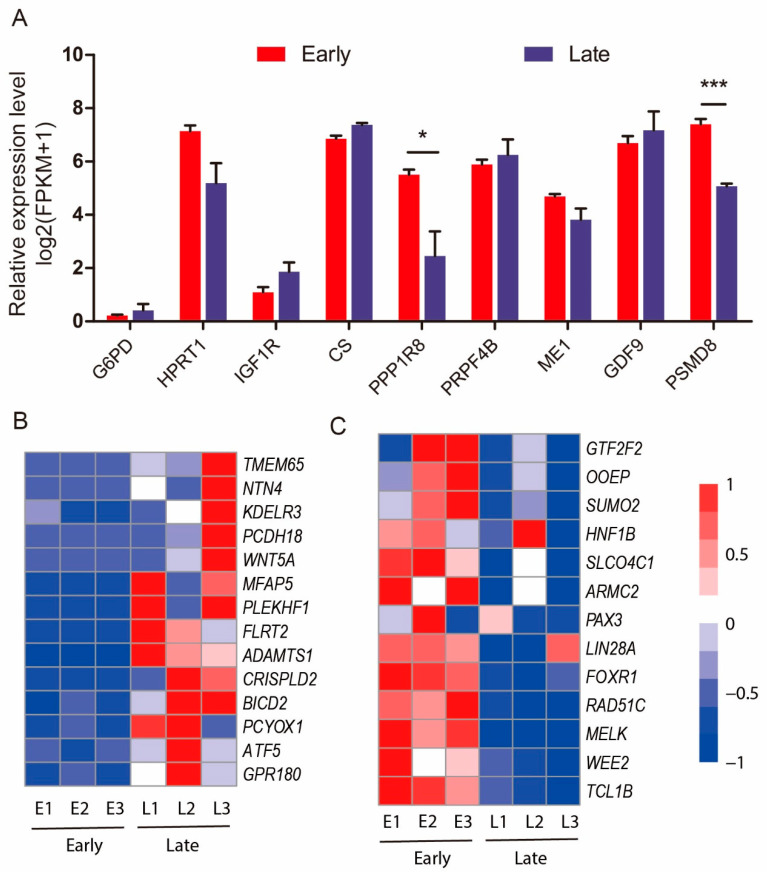
Oocyte-quality-related genes and transcriptional memory genes in pig SCNT reprogramming. (**A**) Relative expression level (log2 (FPKM + 1)) of several oocyte-quality-related genes. * *p* < 0.05, *** *p* < 0.001. (**B**) Heatmap of 14 active memory genes in early-dividing and late-dividing embryos. (**C**) Heatmap of 13 silent memory genes in early-dividing and late-dividing embryos.

Therefore, the expression levels of oocyte-quality-related genes in early-dividing and late-dividing SCNT embryos were compared. Lonergan et al. (2000) reported that 2-cell embryos derived from IVF and cleaved at 27 and 30 h postinsemination had higher levels of mRNA for *G6PD*, *HPRT*, and *IGF1R* than those that cleaved after 33 h [24]. We checked the expression of these genes in early-dividing and late-dividing SCNT embryos, but none of the three genes were expressed differently (Figure 5A). Isom et al. (2012) found that *CS*, *PPP1R8*, *PRPF4B*, *ME1*, *GDF9*, and *PSMD8* were highly expressed in fast-dividing parthenogenetic embryos [11]. However, only *PPP1R8* and *PSMD8* were significantly different between porcine early-dividing and late-dividing SCNT embryos (Figure 5A). These results indicate that in contrast to IVF and parthenogenetic embryos, oocyte quality may not be the primary factor that determines whether an SCNT embryo will develop appropriately.

### 3.6. Somatic Memory Genes in SCNT Embryo Division

Transcriptional memory has been confirmed during SCNT reprogramming [25], and the transcription profile of the donor cells is preserved in SCNT embryos, owing to incomplete reprogramming. Zhou et al. (2020) reported that both abnormal transcriptional activation and transcriptional silencing are involved in the development of bovine SCNT embryos [25]. They summarized 70 abnormally active memory genes and 45 abnormally silent memory genes inherited from donor cells, which were conserved between bovine and mouse reprogramming [25,26]. By analyzing the expression levels of these abnormally active or silent memory genes, we found 14 active memory genes and 13 silent memory genes that were differentially expressed in early- and late-dividing groups. The 14 active memory genes inherited from donor cells are activated in late-dividing SCNT embryos and suppressed in early-dividing embryos. Conversely, the 13 silent memory genes are activated in early-dividing embryos and suppressed in late-dividing embryos. This finding indicated that these abnormally activated or suppressed somatic memory genes were properly reprogrammed in early-dividing embryos. 

Among the 14 active memory genes that are highly expressed in late-dividing SCNT embryos, fibronectin leucine-rich transmembrane protein 2 (*FLRT2)* regulates early embryonic vascular and neural development. Transmembrane protein 65 (*TMEM65)* is a mitochondrial inner-membrane protein; dysfunction of *TMEM65* results in mitochondrial myopathy. Activating transcription factor 5 (*ATF5)* enhances radioresistance and malignancy in cancer cells. The abnormal activation of these genes may hinder the development of embryos. Some EGA-related genes (such as hepatocyte nuclear factor 1-β (*HNF1B*)) and some embryonic development-related genes (such as T-cell leukemia/lymphoma protein 1B (*TCL1B*) and forkhead box R1 (*FOXR1*)) exhibit suppressed transcription in late-dividing embryos, which may impede the development of embryos.

In summary, we found that 14 active memory genes and 13 silent memory genes were properly reprogrammed in early-dividing embryos. This indicates that the nuclear reprogramming of early-dividing SCNT embryos may be more thorough, which may also be the major reason why early-dividing SCNT embryos have higher developmental competence.

## 4. Discussion

Abundant research findings have suggested that IVF, ICSI, and SCNT embryos and parthenogenetic embryos that cleave relatively earlier are more likely to develop to the blastocyst stage in vitro. As cloning efficiency is extremely low, the identification of SCNT embryos with higher developmental potential is of great importance for pig SCNT embryo transfer. Therefore, it is of great significance to analyze the molecular mechanisms leading to differences in the time of the first cleavage of pig cloned embryos. Considering that the use of single-cell RNA-seq is already well established for the analysis of gene expression in large livestock embryos [24,27,28,29], early-dividing and late-dividing pig SCNT embryos were collected and sequenced in this study.

Our study revealed that early-dividing and late-dividing embryos had distinct RNA profiles and that late-dividing SCNT embryos were more different from each other than the early-dividing embryos. There were 3077 DEGs between early-dividing and later-dividing groups, of which 1896 genes were highly expressed in later-dividing embryos, while only 1181 genes were highly expressed in their early dividing counterparts. GO and KEGG analyses revealed that the 1896 DEGs that were highly expressed in later-dividing embryos are enriched in “ribosome”, “mRNA surveillance pathway”, “protein processing in endoplasmic reticulum”, and “spliceosome” pathways. Zhou et al. (2020) found that highly expressed genes in 8-cell bovine SCNT embryos are enriched in “RNA processing”, “translation”, and “ribosome biogenesis” GO terms, which means that compared with 8-cell IVF embryos, the cloned embryos exhibit excessive transcription in RNA processing and translation-related genes and these genes are also significantly higher expressed in donor cells. However, Liu et al. (2018) compared the transcriptome of mouse *in vivo* and SCNT embryos and found that 339 translation-initiation-related genes were downregulated and 1327 transcription-related genes were upregulated in mouse zygote to 2-cell stage SCNT embryos [30]. 

Next, we attempted to determine the major mechanistic components responsible for the first cleavage time of SCNT embryos. Considering that the oocytes used in this study were *in-vitro*-matured and some oocytes mature faster than others, there is a possibility that the first cleavage time of SCNT embryos depends on the extent of ooplasmic maturation. Perhaps the early-cleaving SCNT embryos resulted from oocytes that had matured earlier. As this phenomenon is also true in IVF, ICSI, and parthenogenetic embryos, it has been suggested that the major mechanistic components responsible for developmental potential are the intrinsic quality of oocytes.

Therefore, we compared the expression levels of nine oocyte-quality-related genes [11,24] from early-dividing and late-dividing embryos. The results showed that only two of them were differentially expressed in early- and late-dividing embryos. These results were opposite to the results of IVF [24] and parthenogenetic embryos [11]. Considering the fact that marker genes that can reflect the quality of oocytes are not well identified and validated, it is too early to say that oocyte quality did not play a major role in the first cleavage time of pig SCNT embryos. More information on oocyte-quality-related genes will be helpful to fully characterize this issue. 

Second, recent studies have reported that the activation protocol affects the timing of the first cleavage and the subsequent in vitro developmental potential of SCNT embryos [31]. Combining the findings that the type of donor nucleus used for nuclear transfer affects the timing of the first cleavage [32] and that the donor cell gnome is likely to modify embryo metabolism and physiology as soon as gene transcription begins during the second half of the 1-cell stage [33], it is reasonable to infer that somatic cell nuclear reprogramming may be the main factor that affects the timing of the first cleavage of SCNT embryos. Donor cells from different tissues exhibited variable susceptibility to reprogramming, and donor-cell-specific genes showed abundant overexpression in SCNT embryos [25,27]. As many of the donor-cell-specific genes showed abundant overexpression in SCNT embryos, we hypothesized that there might be more abnormal transcriptional activation and transcriptional silencing in late-dividing SCNT embryos if the late-dividing SCNT embryos have a “worse” reprogramming status than the early-dividing embryos. By compare the FPKM value of abnormally active memory genes and abnormally silent memory genes between the early-dividing and the late-dividing groups, once again, we find that these genes are indeed “worse” reprogrammed in the late-dividing group than in the early-dividing group. In summary, these data demonstrate that reprogramming status might be the major component that determines the developmental competence of porcine SCNT embryos.

In conclusion, this study provides new insights into the global transcriptome of porcine SCNT 2-cell embryos that divide at different times. This knowledge could assist in answering the fundamental question, why do early dividing SCNT embryos have higher developmental potential? However, different research models with meta-analysis approaches are still needed to unravel the full answer to this question. Much work has yet to be done to fully characterize the molecular mechanisms of nuclear reprogramming in order to enhance the efficiency of SCNT embryo production.

## 5. Conclusions

RNA-seq revealed that early-dividing and late-dividing embryos had distinct RNA profiles and that late-dividing SCNT embryos were more different from each other compared with early-dividing embryos. In total, 3077 genes were found to be differentially expressed between early-dividing and later-dividing groups, out of which 1896 genes were highly expressed in later-dividing embryos, while 1181 genes were highly expressed in early-dividing embryos. GO and KEGG analyses revealed that early-dividing embryos have higher expressions of genes that participated in the meiotic cell cycle, while the genes in “ribosome”, “mRNA surveillance pathway”, “protein processing in endoplasmic reticulum”, and “spliceosome” were excessively and highly expressed in late-dividing embryos. The early-dividing SCNT embryos have fewer memory genes that are abnormally activated or suppressed, meaning that the reprogramming of early-dividing SCNT embryos is more thorough, which might be the major component that determines the developmental competence of porcine SCNT embryos.

## Figures and Tables

**Table 1:** *In vitro* development of SCNT embryos with different first division times.

Number of Cultured Embryos	Replicates	Time of First Division	Number of 2-Cell Embryos	Number (%) of Blastocysts *
295	4	12–24 h	108	36 (33.6 ± 3.7) ^a^
		25–36 h	93	11 (11.3 ± 3.5) ^b^

^a,b^ Different superscripts within the same column represent significant differences (*p* < 0.05). * Blastocyst formation refers to the percentage of cleaved embryos that developed into blastocysts; the value is represented as mean ± SD.

## Supplementary Materials

The following are available online at https://www.mdpi.com/2073-4425/11/12/1499/s1. Figure S1: Plot of PCA loading. Table S1: QC summary. Table S2: Mapping statistics. Table S3: All gene FPKM values. Table S4: Retained genes that fall within the top and bottom 1% fractions of the PC1 and PC2 loadings’ range.

## References

[1] Gouveia C., Huyser C., Egli D., Pepper M.S. (2020). Lessons learned from somatic cell nuclear transfer. Int. J. Mol. Sci..

[2] Wang X., Qu J., Li J., He H., Liu Z., Huan Y. (2020). Epigenetic reprogramming during somatic cell nucleartTransfer: Recent progress and future Directions. Front. Genet..

[3] Ochota M., Niżański W. (2017). Time of early cleavage affects the developmental potential of feline preimplantation embryos in vitro. Theriogenology.

[4] Luo X., Xiao W., Feng C., Long C., Yan J., Xue Z., Yun P., Pan D. (2010). Timing of the first Zyogte cleavage as a developmental potential marker for procine cloned Embryos. Prog. Biochem. Biophys..

[5] Lundin K., Bergh C., Hardarson T. (2001). Early embryo cleavage is a strong indicator of embryo quality in human IVF. Hum. Reprod..

[6] Bos-Mikich A., Mattos A.L.G., Ferrai A.N. (2001). Early cleavage of human embryos: An effective method for predicting successful IVF/ICSI outcome. Hum. Reprod..

[7] Lechniak D., Pers-Kamczyc E., Pawlak P. (2008). Timing of the first zygotic cleavage as a marker of developmental potential of mammalian embryos. Reprod. Biol..

[8] Lee M.J., Lee R.K., Lin M.H., Hwu Y.M. (2012). Cleavage speed and implantation potential of early-cleavage embryos in IVF or ICSI cycles. J. Assist. Reprod. Genet..

[9] Milazzotto M.P., Goissis M.D., Chitwood J.L., Annes K., Soares C.A., Ispada J., Assumpção M.E.O.Á., Ross P.J. (2016). Early cleavages influence the molecular and the metabolic pattern of individually cultured bovine blastocysts. Mol. Reprod. Dev..

[10] Torner E., Bussalleu E., Briz M.D., Yeste M., Bonet S. (2013). Energy substrate influences the effect of the timing of the first embryonic cleavage on the development of in vitro-produced porcine embryos in a sex-related manner. Mol. Reprod. Dev..

[11] Isom S.C., Li R.F., Whitworth K.M., Prather R.S. (2012). Timing of first embryonic cleavage is a positive indicator of the in vitro developmental potential of porcine embryos derived from in vitro fertilization, somatic cell nuclear transfer and parthenogenesis. Mol. Reprod. Dev..

[12] Lee Y.S., Thouas G.A., Gardner D.K. (2015). Developmental kinetics of cleavage stage mouse embryos are related to their subsequent carbohydrate and amino acid utilization at the blastocyst stage. Hum. Reprod..

[13] Kobayashi T., Kato Y., Tsunoda Y. (2004). Effect of the timing of the first cleavage on the developmental potential of nuclear-transferred mouse oocytes receiving embryonic stem cells. Theriogenology.

[14] Son Y.J., Lee S.E., Park Y.G., Jeong S.G., Shin M.Y., Kim E.Y., Park S.P. (2018). Fibroblast growth factor 10 enhances the developmental efficiency of Somatic Cell Nuclear Transfer Embryos by Accelerating the Kinetics of Cleavage During *In Vitro* Maturation. Cell. Reprogramming.

[15] Lai L., Prather R.S. (2003). Production of cloned pigs by using somatic cells as donors. Cloning Stem Cells.

[16] Wei Y., Fan J., Li L., Liu Z., Li K. (2016). Pretreating porcine sperm with lipase enhances developmental competence of embryos produced by intracytoplasmic sperm injection. Zygote.

[17] Yoshioka K., Suzuki C., Tanaka A., Anas I.M.-K., Iwamura S. (2002). Birth of piglets derived from porcine zygotes cultured in a chemically defined medium1. Biol. Reprod..

[18] Dobin A., Davis C.A., Schlesinger F., Drenkow J., Zaleski C., Jha S., Batut P., Chaisson M., Gingeras T.R. (2013). STAR: Ultrafast universal RNA-seq aligner. Bioinformatics.

[19] Trapnell C., Williams B.A., Pertea G., Mortazavi A., Kwan G., van Baren M.J., Salzberg S.L., Wold B.J., Pachter L. (2010). Transcript assembly and quantification by RNA-Seq reveals unannotated transcripts and isoform switching during cell differentiation. Nat. Biotechnol..

[20] Liao Y., Wang J., Jaehnig E.J., Shi Z., Zhang B. (2019). WebGestalt 2019: Gene set analysis toolkit with revamped UIs and APIs. Nucleic Acids Res..

[21] ENCODE Project Consortium (2012). An integrated encyclopedia of DNA elements in the human genome. Nature.

[22] Nakai M., Ozawa M., Maedomari N., Noguchi J., Kaneko H., Ito J., Onishi A., Kashiwazaki N., Kikuchi K. (2014). Delay in cleavage of porcine embryos after intracytoplasmic sperm injection (ICSI) shows poorer embryonic development. J. Reprod. Dev..

[23] Jeon Y., Jeong S.H., Biswas D., Jung E.M., Jeung E.B., Lee E.S., Hyun S.H. (2011). Cleavage pattern and survivin expression in porcine embryos by somatic cell nuclear transfer. Theriogenology.

[24] Lonergan P., Gutiérrez-Adán A., Pintado B., Fair T., Ward F., Fuente J.D., Boland M. (2000). Relationship between time of first cleavage and the expression of IGF-I growth factor, its receptor, and two housekeeping genes in bovine two-cell embryos and blastocysts produced in vitro. Mol. Reprod. Dev..

[25] Zhou C., Zhang J., Zhang M., Wang D., Ma Y., Wang Y., Wang Y., Huang Y., Zhang Y. (2020). Transcriptional memory inherited from donor cells is a developmental defect of bovine cloned embryos. FASEB J. Off. Publ. Fed. Am. Soc. Exp. Biol..

[26] Kim K., Doi A., Wen B., Ng K., Zhao R., Cahan P., Kim J., Aryee M.J., Ji H., Ehrlich L.I.R. (2010). Epigenetic memory in induced pluripotent stem cells. Nature.

[27] Zhang L., Yu M., Xu H., Wei X., Liu Y., Huang C., Chen H., Guo Z. (2020). RNA sequencing revealed the abnormal transcriptional profile in cloned bovine embryos. Int. J. Biol. Macromol..

[28] Li H., Song M., Yang W., Cao P., Zheng L., Zuo Y. (2020). A comparative analysis of single-cell transcriptome identifies reprogramming driver factors for efficiency improvement. Mol. Ther. Nucleic Acids.

[29] Duan J., Zhu L., Dong H., Zheng X., Jiang Z., Chen J., Tian X.C. (2019). Analysis of mRNA abundance for histone variants, histone- and DNA-modifiers in bovine *in vivo* and in vitro oocytes and embryos. Sci. Rep..

[30] Liu Y., Wu F., Zhang L., Wu X., Li D., Xin J., Xie J., Kong F., Wang W., Wu Q. (2018). Transcriptional defects and reprogramming barriers in somatic cell nuclear reprogramming as revealed by single-embryo RNA sequencing. BMC Genomics.

[31] Akagi S., Tamura S., Matsukawa K. (2020). Timing of the first cleavage and in vitro developmental potential of bovine somatic cell nuclear transfer embryos activated by different protocols. Cell. Reprogramming.

[32] Amarnath D., Kato Y., Tsunoda Y. (2007). Effect of the timing of first cleavage on in vitro developmental potential of nuclear-transferred bovine oocytes receiving cumulus and fibroblast cells. J. Reprod. Dev..

[33] Gao S., Latham K.E. (2004). Maternal and environmental factors in early cloned embryo development. Cytogenet. Genome Res..

