# Supplementary material for: Transcriptome Analyses Reveal Differential Transcriptional Profiles in Early- and Late-Dividing Porcine Somatic Cell Nuclear Transfer Embryos
Source: Genes (Basel). 2020 Dec 12;11(12):1499. doi: 10.3390/genes11121499 (PMC7763450; doi:10.3390/genes11121499)
Supplement: Supplementary file 1 [file genes-11-01499-s001.zip › FigureS1_TableS4.PCA_loading_and_retained_genes.docx]

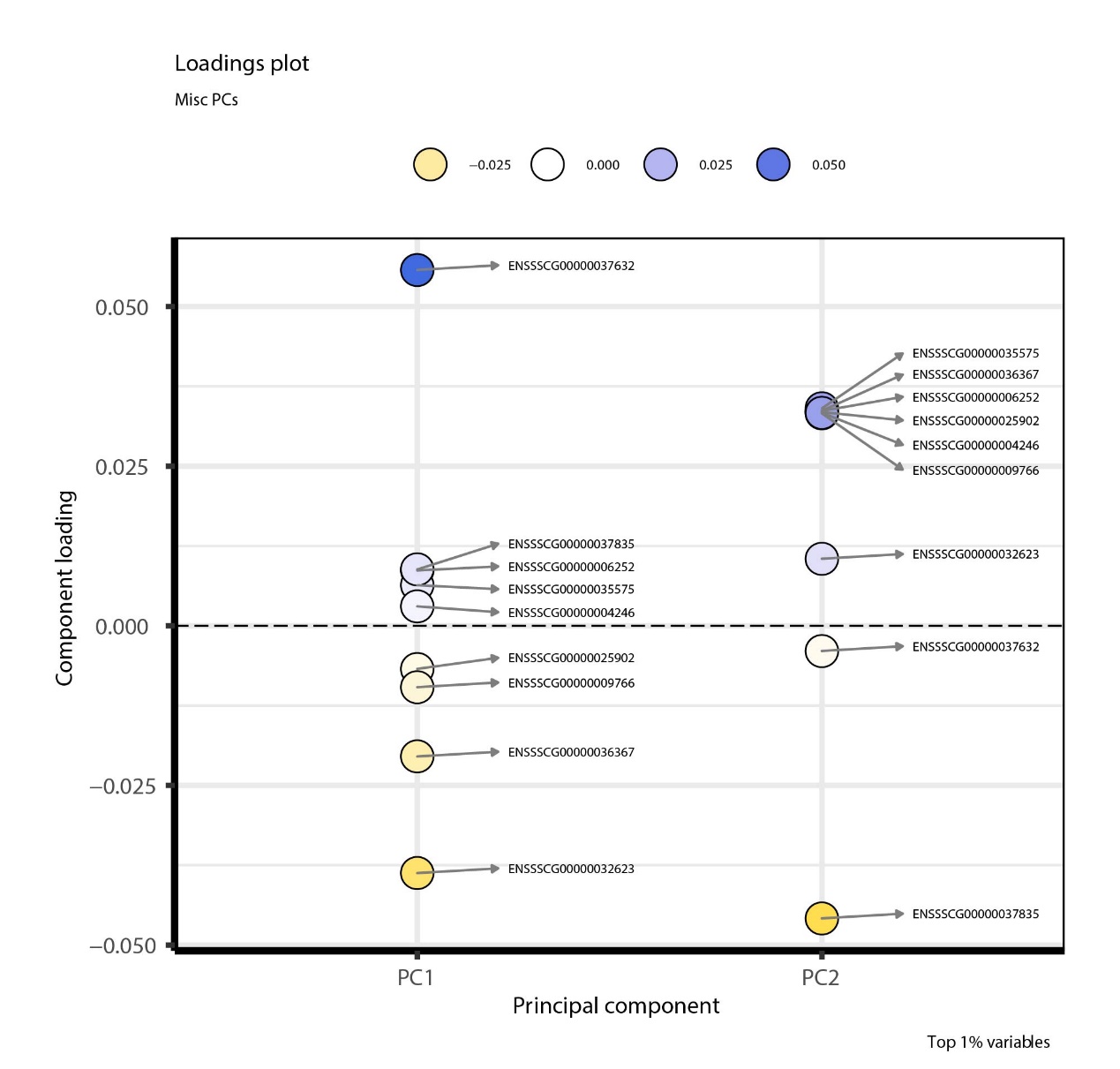


**Figure S1 Plot of PCA loading.**

Table S4 Retained genes that fall within the top and bottom 1% fraction of the PC1 and PC2 loadings range

| Ensembl_ID | Gene_symbol | Early-cleaving | Late-cleaving | log2FoldChange | FDR | Descriptions |
| --- | --- | --- | --- | --- | --- | --- |
| ENSSSCG00000037632 | [LOC100625127](http://www.ncbi.nlm.nih.gov/entrez/query.fcgi?db=gene&cmd=Retrieve&dopt=Graphics&list_uids=100625127) | 0 | 11261.94 | -16.02 | 2.1E-23 | melanoma antigen preferentially expressed in tumors-like |
| ENSSSCG00000032623 | [NA](https://biodbnet-abcc.ncifcrf.gov/dbInfo/faq.php#data5) | 876.66 | 0.26 | 11.26 | 1.3E-15 |  |
| ENSSSCG00000036367 | [PABPN1L](http://www.ncbi.nlm.nih.gov/sites/entrez?db=gene&term=PABPN1L) | 4280.38 | 307.67 | 3.80 | 0.04047 | PABPN1 like, cytoplasmic |
| ENSSSCG00000006252 | [TMEM68](http://www.ncbi.nlm.nih.gov/sites/entrez?db=gene&term=TMEM68) | 122.35 | 1476.89 | -3.59 | 0.09156 | transmembrane protein 68 |
| ENSSSCG00000035575 | [LOC110261380](http://www.ncbi.nlm.nih.gov/sites/entrez?db=gene&term=LOC110261380) | 165.23 | 1258.83 | -2.93 | 0.17506 |  |
| ENSSSCG00000004246 | [NA](https://biodbnet-abcc.ncifcrf.gov/dbInfo/faq.php#data5) | 234.31 | 942.87 | -2.01 | 0.37079 |  |
| ENSSSCG00000009766 | [ATP6V0A2](http://www.ncbi.nlm.nih.gov/sites/entrez?db=gene&term=ATP6V0A2) | 1085.39 | 511.18 | 1.09 | 0.66322 | ATPase H+ transporting V0 subunit a2 |
| ENSSSCG00000037835 | [TRIM7](http://www.ncbi.nlm.nih.gov/sites/entrez?db=gene&term=TRIM7) | 0.38 | 259.45 | -9.49 | 1 | tripartite motif containing 7 |
| ENSSSCG00000025902 | [ANAPC4](http://www.ncbi.nlm.nih.gov/sites/entrez?db=gene&term=ANAPC4) | 598.92 | 863.89 | -0.53 | 1 | anaphase promoting complex subunit 4 |
